# Reconstructing Missing and Anomalous Data Collected from High-Frequency In-Situ Sensors in Fresh Waters

**DOI:** 10.3390/ijerph182312803

**Published:** 2021-12-04

**Authors:** Claire Kermorvant, Benoit Liquet, Guy Litt, Jeremy B. Jones, Kerrie Mengersen, Erin E. Peterson, Rob J. Hyndman, Catherine Leigh

**Affiliations:** 1 Laboratoire de Mathématiques et de Leurs Applications de Pau Fédération MIRA, UMR CNRS 5142, Université de Pau et des Pays de l’Adour, 64600 Anglet, France; benoit.liquet-weiland@mq.edu.au; 2 Department of Mathematics and Statistics, Macquarie University, Sydney, NSW 2109, Australia; 3 National Ecological Observatory Network, Battelle Boulder, Boulder, CO 80301, USA; glitt@battelleecology.org; 4 Institute of Arctic Biology and Department of Biology and Wildlife, University of Alaska Fairbanks, Fairbanks, AK 99775, USA; jbjonesjr@alaska.edu; 5 School of Mathematical Sciences, Queensland University of Technology, Brisbane, QLD 4000, Australia; k.mengersen@qut.edu.au; 6 ARC Centre of Excellence for Mathematics and Statistical Frontiers, Melbourne, VIC 3000, Australia; erin@peterson-consulting.com (E.E.P.); rob.hyndman@monash.edu (R.J.H.); catherine.leigh@rmit.edu.au (C.L.); 7 Peterson Consulting, Brisbane, QLD 4000, Australia; 8 Department of Econometrics and Business Statistics, Monash University, Clayton, VIC 3800, Australia; 9 Biosciences and Food Technology Discipline, School of Science, RMIT University, Bundoora, VIC 3083, Australia

**Keywords:** anomaly correction, generalised additive model (GAM), missing data reconstruction, remote sensing, water quality

## Abstract

In situ sensors that collect high-frequency data are used increasingly to monitor aquatic environments. These sensors are prone to technical errors, resulting in unrecorded observations and/or anomalous values that are subsequently removed and create gaps in time series data. We present a framework based on generalized additive and auto-regressive models to recover these missing data. To mimic sporadically missing (i) single observations and (ii) periods of contiguous observations, we randomly removed (i) point data and (ii) day- and week-long sequences of data from a two-year time series of nitrate concentration data collected from Arikaree River, USA, where synoptically collected water temperature, turbidity, conductance, elevation, and dissolved oxygen data were available. In 72% of cases with missing point data, predicted values were within the sensor precision interval of the original value, although predictive ability declined when sequences of missing data occurred. Precision also depended on the availability of other water quality covariates. When covariates were available, even a sudden, event-based peak in nitrate concentration was reconstructed well. By providing a promising method for accurate prediction of missing data, the utility and confidence in summary statistics and statistical trends will increase, thereby assisting the effective monitoring and management of fresh waters and other at-risk ecosystems.

## 1. Introduction

Water quality sampling and analysis commonly relies on manual approaches, such as grab sampling and laboratory analyses, often conducted at monthly or longer intervals for variables such as sediment and nutrient concentration [1]. As such, the ability to capture water quality events or determine patterns and trends at fine spatial and temporal resolution are often limited [2]. Advances in the development of in situ, high-frequency environmental sensors have led to their expanded use in environmental monitoring, including for fresh waters [3]. As the cost-effectiveness and telecommunications capability of these sensors increases, their ability to provide high-frequency data in near real time likewise increases, allowing managers and decision makers to act in a timelier and more spatially specific fashion. The large datasets generated by high-frequency in situ sensors also present new opportunities for scientists when analysing, modelling, and reporting water quality data [4,5]. Consequently, high-frequency datasets collected from in situ sensors can provide a more thorough understanding of water quality dynamics at multiple time scales and help to improve data quality assurance and quality control [6].

In situ sensors, despite their benefits, are prone to technical errors due to biofouling, power failures, and other issues. These errors can lead to technical anomalies in water quality data and potentially confound the assessment or identification of true changes in water chemistry [7]. Given that the high frequency and large size of these datasets precludes the use of manual anomaly detection methods (one part of the entire data quality assurance and quality control process), various automated approaches have been proposed. For example, Shi et al. (2018) [8] integrated a wavelet artificial neural network with surrogate measurements for rapid warning of water quality anomalies, Liu et al. (2020) [9] integrated a Bayesian autoregressive model with an Isolation Forest algorithm for combined prediction and detection, while Rodriguez-Perez et al. (2020) [10] developed a semi-supervised Bayesian artificial neural network approach. To assist both developers and end users, Leigh et al. (2019) [7] developed a ten-step anomaly detection framework to systematically implement and compare suites of anomaly detection methods based on end-user needs.

Regardless of the method used to detect water quality anomalies from in situ sensors, observations that get labelled as anomalous are often subsequently removed from the time series, rendering them missing. Furthermore, given the variety of types of technical anomalies, such as sudden spikes, unrealistic values, drift, or periods of anomalously high or low variability [7], the resultant time series may contain missing point observations and/or sequences of contiguously missing observations after the data are passed through an anomaly detection algorithm. Failure to replace anomalies with corrected data may occur because methods to confidently reconstruct (accurately predict) the true values of the missing water quality observations are not available. Missing data then create data quality issues [11] and can lead to biased estimates of parameters, increased standard errors, decreased statistical power, and lost information [12], which may hinder the calculation of summary statistics [13] and affect statistical trend detection [14]. Slater et al., 2017 [15], for example, demonstrated via simulation that the loss in trend detection tends to increase with an increasing size of missing data ‘gaps’ and decreasing length of time series.

Many of the commonly used methods used to reconstruct missing water quality data were developed before the proliferation of high-frequency sensors, such as infilling based on surrounding data [16,17], regression analysis [18,19], state–space models with an estimation maximization algorithm [20], or artificial neural networks [21,22,23], and, therefore, were targeted at data with lower-frequency time steps, such as daily data. More recently, but also based on daily data, various infilling techniques, such as regression, scaling, and equi-percentile approaches [24], along with dynamic regression models [25], have been used to reconstruct missing streamflow data. Methods developed in other domains, such as computer science, have reconstructed missing sensor data based on temporal or spatial correlation, interpolation, and sparse theory [26]. In sensor networks, linear and non-linear regression methods have been developed that use the non-missing data adjacent to the missing data [27], along with algorithms based on combining K-means algorithms and neural networks with particle swarm optimization [28]. Similar methods have been developed in other domains, such as those used for power systems within computer science [29], while in the engineering domain, bidirectional recurrent neural networks have been developed to reconstruct sensor data used to monitor bridge construction [30]. As can be seen, these various methods of data reconstruction have been developed fit for purpose and as solutions for domain-specific problems. Hence, we aimed to develop a suitable method to reconstruct high-frequency nutrient data collected from in situ sensors in rivers, a problem, to our knowledge, that is yet to be addressed.

In the environmental domain, and specifically river management, nutrient monitoring, and specifically that of nitrate concentration, is particularly important. In its bio-available form, nitrate is assimilated for growth and metabolism by riverine biota (e.g., algae, macrophytes, and some bacteria) that form the basal components of aquatic food webs [31]. However, an excess of nitrate can lead to problems like eutrophication, leading to a decrease in light infiltration and dissolved oxygen concentration [32,33], which, in turn, can negatively affect the health of aquatic biota such as fishes and invertebrates [34,35,36], as well as increasing costs for water treatment and complicating management of river ecosystems spanning catchment headwaters to receiving waters downstream, including oceans [37]. Furthermore, nitrate concentration can vary substantially in space and time in river ecosystems due to instream processes and external inputs [38,39]. This high spatial and temporal variation has increased the interest in and use of high-frequency, in situ nitrate sensors in river monitoring programs and, thus, the need to develop appropriate methods to reconstruct missing nitrate concentration data from the resulting time series.

While it is important to develop a sound method to confidently reconstruct missing nitrate data for use in environmental management, the use of nitrate data can also serve as a case study to demonstrate the potential for the method to be applied more broadly. As such, the objective of this study was to develop and test a data reconstruction method using both a real time series of high-frequency nitrate concentrations and a simulation study.

## 2. Materials and Methods

### 2.1. Reconstruction Method

Let *Y* be the response (i.e., dependent) variable of interest and *Y_t_* the value taken by *Y* at time *t*. For covariates *X* ∈m (i.e., *m* explanatory variables or predictors) we denoted *X_kt_*, the *k*th covariates observed at time *t*. We then identified two possible cases: (i) all *X_kt_* are available at the same time step as the variable of interest *Y_t_*, and (ii) at least one covariate is not available at time *t*. For the first case, when all *X_kt_* were available, we used a generalised additive model to predict *Y* at each time *t* that was missing, following the equation:

(1) $$Y_{t}=\beta_{0}+\overset{m}{\underset{k=1}{\sum }}s_{k}\left(X_{kt}\right)+\varepsilon_{t}$$

where *X_kt_* are covariates measured at the *t*th sample. Here, *β*_0_ is an intercept and *ε_t_* is an error term, following the usual i.i.d assumptions that we make about regression errors, *ε_t_* ~ *N*(0, *σ*^2^). The associated smooth function *s_k_*(·) of each water quality variable *X_k_* was defined using thin plate spline regression [40]. A forwards and backwards stepwise variable selection procedure was implemented and the ’best’ GAM model (in terms of variables selected and penalisation of smooth splines) was identified based on the Akaike Information Criterion (AIC) [41].

**Figure 1:**
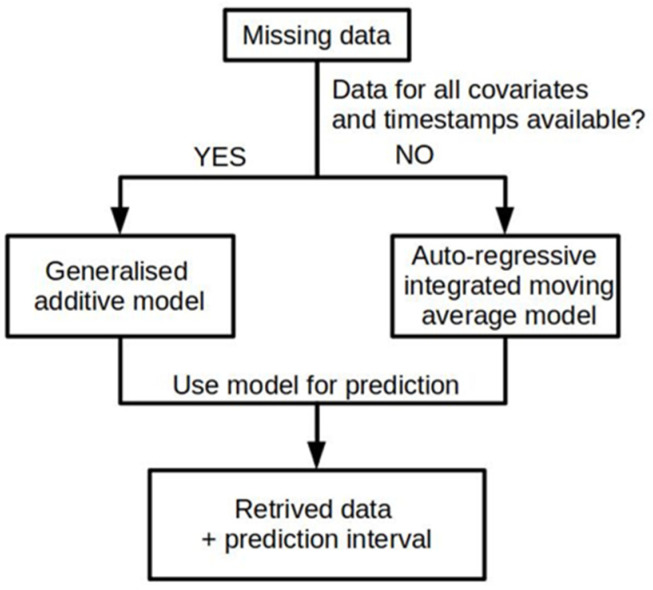
Reconstruction method. Flow chart of the method to predict the values of missing observations in high-frequency sensor data.

For the case when at least one covariate was not available at time *t*, such that *Y_t_* could not been predicted with the GAM model, we used an autoregressive integrated moving average model (ARIMA, Figure 1) [42]. For each missing *Y_t_* that could not be predicted with a GAM, we used the 500 previous *Y* observations (*Y_t−_*_500_, *Y_t−_*_499_, …, *Y_t−_*_2_, *Y_t−_*_1_) and selected the best ARIMA model by AIC comparisons. Prediction intervals (95%) associated with the reconstructed values were then calculated according to the model used.

### 2.2. Arikaree River Case Study: Applying the Reconstruction Method

We applied the missing-data reconstruction method to time series of water-quality data collected from Arikaree River, a small wadeable stream in the semi-arid eastern Colorado plains of the United States of America. The Arikaree River site has a catchment of 2632 km^2^, comprising mainly grasslands and irrigated agricultural land, and is part of the National Ecological Observatory Network (NEON). NEON collects and provides open data from aquatic and terrestrial sites across the United States of America (USA), including data from high-frequency, in situ sensors. NEON conducts standardised configuration, calibration, and preventive maintenance procedures on all their sensors [43,44] and follows in situ measurement and sample analysis protocols as outlined in [45]. As such, the Arikaree River site provided us with a suitable time series of water quality data for the purposes of this study.

Several water quality variables are available from each NEON aquatic site (Table A1). Nitrate concentration [46] is measured in µmol/L using a 10 mm path length SUNA V2 UV light spectrum sensor. The SUNA V2 collects data reported as a mean value from 20 measurements made during a sampling burst every 15 min. The published nitrate resolution is 0.1 µmol/L and the manufacturer’s stated sensor accuracy is approximately 2 µmol/L or 10% of the reading above 20 µmol/L. We, therefore, report units of measurement for nitrate in µmol/L (1 µmol nitrate/L = 0.062 mg nitrate/L). Other co-located sensors report specific conductance (µS/cm), dissolved oxygen (mg/L), water temperature (°C), and turbidity (Formazin Nephelometric Units, FNU) data as one-minute instantaneous measurements [47,48]. Water elevation data (i.e., water level as meters above sea level) are published as five-minute averaged measurements from data sampled at 1 min intervals [49]. 

**Figure 2:**
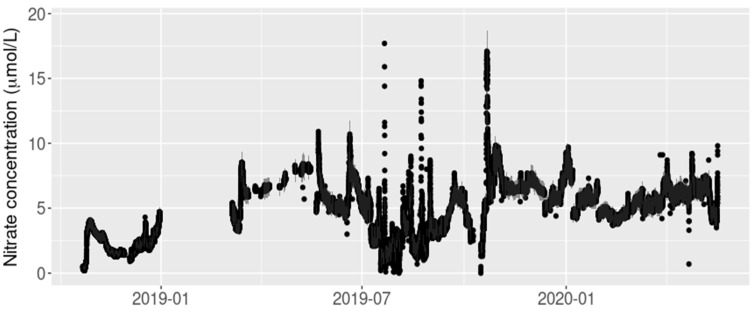
Arikaree River nitrate data. Black points represent the original nitrate observations, grey shading represents the precision interval of the sensor.

For this study, we used a two-year period of nitrate concentration data from one of two sites (the downstream site) on the Arikaree River, from October 2018 to October 2020 (*n* = 73,056 nitrate concentration observations), in which there were already missing point data and missing periods of data (14,283 missing observations of nitrate concentration = 20% of the nitrate data in total) (Figure 2). These data had all been removed from the time series as part of the NEON data quality assurance and quality control process. For example, there was a technical issue with the nitrate sensor during winter 2019 so no nitrate concentration measurements were available for the first three months of 2019. Missing data were also present in the time series of the covariates as a result of quality control and assurance processing: 29% of the temperature time series, 35% of the specific conductance time series, 14% of the dissolved oxygen time series, 47% of the turbidity time series, and 11% of the elevation time series.

We considered nitrate concentration as our *Y* and the other water quality variables (specific conductance, dissolved oxygen, water temperature, turbidity, and water elevation) as the covariates *X* that could be related to *Y* [50]. Visual examination of the distributions of the response and covariates indicated that turbidity had a strongly right-skewed distribution and was, therefore, log-transformed (i.e., log (turbidity + 1)) prior to analysis [51]. We also included two additional covariates to account for temporal autocorrelation in the time series, as determined by the AIC. The first additional covariate was nitrate concentration at one time step before time *t* (i.e., *Y_t−_*_1_) and the second was nitrate concentration at two time steps before time *t* (i.e., *Y_t−_*_2_). 

### 2.3. Simulation Study: Performance Evaluation

To evaluate the performance of our reconstruction method, we then repeatedly and randomly removed different combinations of data (both point observations and sequences of contiguous observations) from the two-year time series of nitrate concentration from Arikaree River. For the missing point data, we randomly removed 20%, 30%, and 40% of the observations from the nitrate concentration time series and repeated this process 100 times each (Simulations 1, 2, and 3). For the missing sequences of data, we randomly removed ten individual days (10 × 24 h worth) of observations, repeating the process 100 times (Simulation 4), as well as ten individual weeks of observations, again repeating the process 100 times (Simulation 5). 

For each simulation, we then calculated the root-mean-square error (RMSE) and the proportion of reconstructed data within the precision interval of the nitrate sensor (PWPI), i.e., ±10% for readings > 20 µmol/L and ±2 µmol/L for readings < 20 µmol/L.

### 2.4. Implementation

Simulation and imputation were performed with the base packages within the R statistical software [52]. Modelling was undertaken using the car [53], gam [54], mgcv [39], and forecast [55] packages. The R script used to implement the analyses is provided in the GitHub repository available online at https://github.com/Claire-K/nitrate_time_serie_reconstruction (accessed on 4 December 2021) and the Arikaree data are available from NEON [46,47,48,49] (see Table A1 for data product numbers).

## 3. Results

### 3.1. Arikaree River Case Study

**Figure 3:**
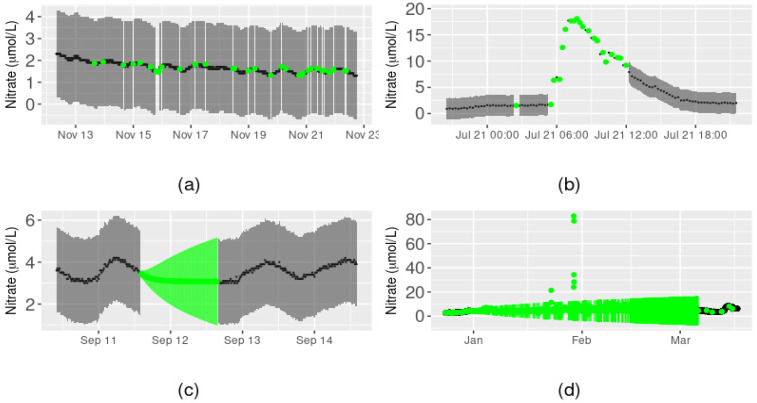
Reconstruction of Arikaree nitrate data. Green points represent the nitrate concentration values predicted by the reconstruction method, along with intervals of prediction, for (**a**) a 12-day sequence of sporadically missing data, (**b**) a one-day peak flow event containing sporadically missing data, (**c**) a full day of contiguously missing data, and (**d**) a three-month sequence of contiguously missing data. Black points represent the original nitrate observations, grey shading represents the precision interval of the sensor. Prediction intervals may not be visible when they are narrow relative to the precision interval of the sensor.

Model performance varied according to the characteristics of the missing data. For demonstration purposes, we focus here on four different cases: (a) a 12-day sequence in which data were sporadically missing, (b) a one-day peak flow event containing sporadically missing data, (c) a full day of missing data, and (d) a three-month sequence of missing data (Figure 3). Our method performed well at predicting values of nitrate concentration where point observations were sporadically missing from the time series. In other words, the predicted values followed the pattern of the surrounding data closely and prediction intervals were narrow compared to the sensor precision interval (e.g., Figure 3a,b).

However, the method performed less well when periods of contiguously missing observations were reconstructed. For the single day of missing data, the daily pattern in nitrate concentration present in the surrounding data was not reconstructed, and the prediction interval of the reconstructed nitrate values increased with the number of missing observations (Figure 3c). This was also the case for the reconstruction of the three-month period of missing data (Figure 3d). However, some extremely high nitrate concentrations (~80 µmol/L) were predicted to occur during this period, based on the values of the covariates at the time, which had not been detected as anomalous by the data quality assurance and control process [56]. This demonstrated that the quality of the reconstructed data can depend heavily on the covariates, when available, and therefore, reliable performance of any anomaly detection method implemented prior to reconstruction is crucial. 

We also found that reconstructed values of nitrate had much larger prediction intervals when ARIMA, rather than GAM, was used due to the presence of missing data in the covariate(s), which simply by chance would more often occur during contiguous sequences of missing nitrate data than during periods of similar length with sporadically missing nitrate data. Overall, the prediction interval for the 14,283 missing values in the nitrate time series ranged from 0.01 to 56.03 µmol/L, with a median of 1.34 µmol/L.

### 3.2. Simulation Study: Performance Evaluation

#### 3.2.1. Simulations 1, 2, and 3: Missing Point Data

**Figure 4:**
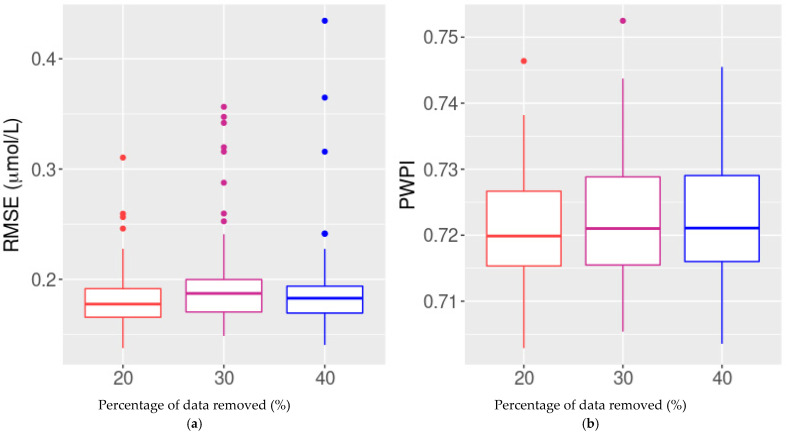
Performance evaluation: reconstructing missing point data. Boxplots of (**a**) root-mean-square error (RMSE) and (**b**) the proportion of reconstructed data within the precision interval (PWPI) for different amounts of randomly removed point observations, simulated 100 times.

In terms of the reconstruction performance of our method, the RMSE values from simulations 1, 2, and 3 (20%, 30% and 40% of randomly missing point data in the nitrate time series, respectively) were all similar and rarely >0.2 µmol/L, even with 40% of the data having been removed (Figure 4a). Furthermore, the method predicted more than 95% of the missing nitrate values with an RMSE of 0.2 µmol/L. Nevertheless, as the proportion of missing data increased, so did the maximum RMSE. Overall, 72% of the reconstructed nitrate values were within the precision interval of the sensors (Figure 4b).

**Figure 5:**
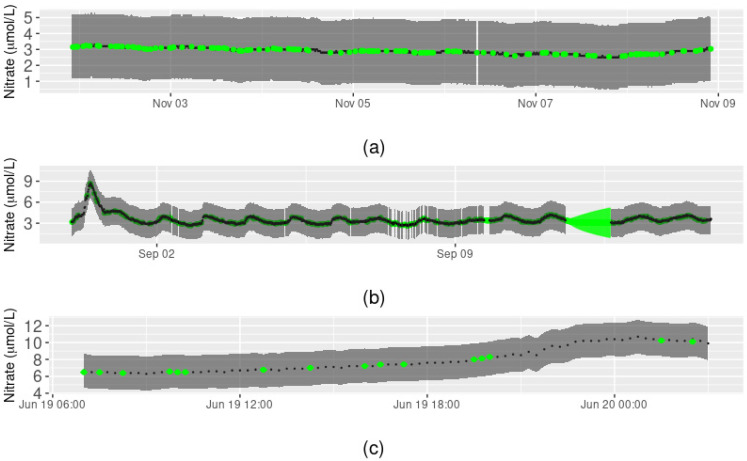
Performance evaluation: missing point data examples. Examples for different periods of randomly removed point observations, simulated 100 times: (**a**) one week, (**b**) one month, and (**c**) a nitrate event in which concentrations rose rapidly in less than 24 h. Dark points represent the real nitrate concentration value and the grey shading around those points represents the precision interval of the sensor. Green points and shading are the predicted values along with the prediction interval. Prediction intervals may not be visible when they are narrow relative to the precision interval of the sensor.

The performance of our method in reconstructing missing point data can also be demonstrated by looking more closely at different periods of the simulated Arikaree River time series, including typical baseflow and storm event behaviours of nitrate concentration. In all cases, the predicted data followed the pattern of nitrate concentration closely, including a peak event that occurred over a period of less than 24 h (Figure 5).

#### 3.2.2. Simulations 4 and 5: Missing Sequences of Data

**Figure 6:**
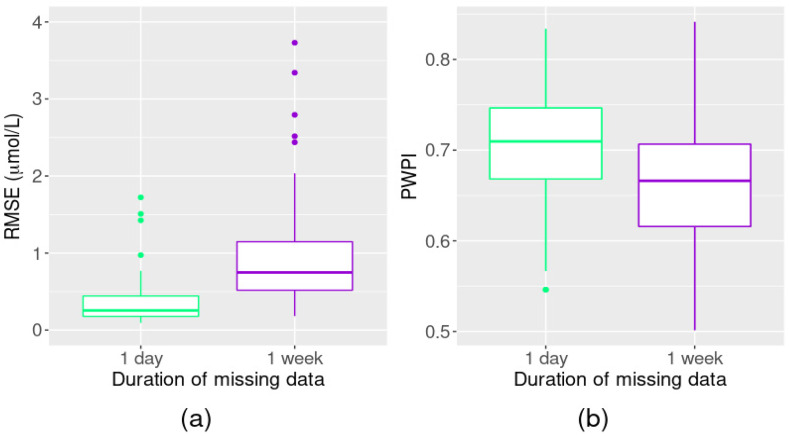
Performance evaluation: reconstructing sequences of missing data. Boxplots of (**a**) root-mean-square error (RMSE) and (**b**) the proportion of reconstructed data within the precision interval (PWPI) for different amounts of randomly removed point observations, simulated 100 times. Note that the *y*-axis on plot (**a**) has been truncated at 4 µmol/L (one extreme RMSE value of 45.23 µmol/L for the one-week simulations not shown).

When reconstructing missing sequences of data in the simulated time series, performance declined as sequence duration increased (i.e., the RMSE increased and PWPI decreased (Figure 6). The median and third quartile of RMSE for simulations where 10 day-long sequences of data were randomly removed were 0.25 µmol/L and 0.44 µmol/L, respectively, compared with 0.75 µmol/L and 1.16 µmol/L, respectively, for simulations where 10 week-long sequences were randomly removed. For the median and third quartile PWPI, the one-day vs. one-week comparisons were 0.70 and 0.74 vs. 0.66 and 0.70, respectively.

**Figure 7:**
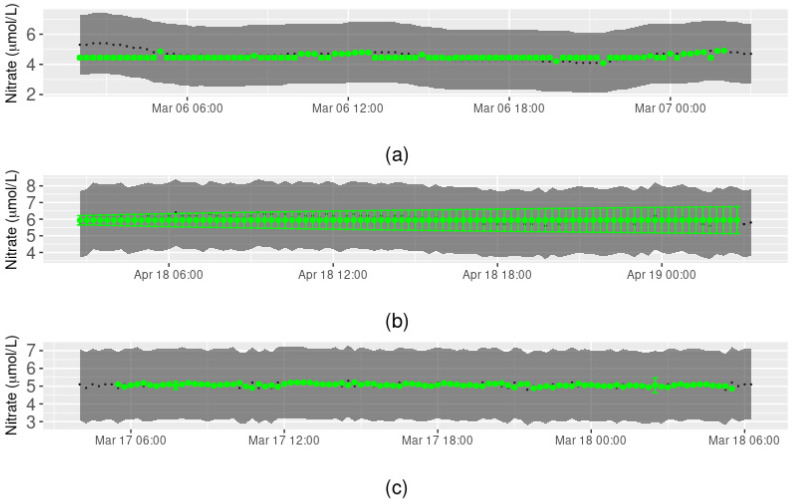
Performance evaluation: missing sequential data examples. Examples for different periods of randomly removed one-week sequences of observations, simulated 100 times, where data were reconstructed using (**a**) GAM and ARIMA, (**b**) ARIMA only, and (**c**) GAM only. Dark points represent the real nitrate concentration value and the shaded area around those points is the precision interval of the sensor. Green points and shading are the predicted values along with the prediction interval. Prediction intervals may not be visible when they are narrow relative to the precision interval of the sensor.

We also observed that performance depended on whether GAM and ARIMA, GAM alone, or ARIMA alone was used for the reconstruction. When ARIMA was used, the amount of missing data present in the preceding period also impacted performance. For example, both GAM and ARIMA were required for a week-long reconstruction in early March 2019 (Figure 7a), but this week occurred just after a three-month period of missing data, such that the ARIMA (based on the previous 500 observations) was unable to perform well. The ARIMA always predicted a nitrate concentration of 4.5 µmol/L for missing data in the week-long sequence, whereas the GAM predictions followed the actual concentrations closely. This was also the case when GAM was used alone due to all covariates being available throughout the week-long sequence (Figure 7c).

In the case where ARIMA was used after a period with little to no missing values (Figure 7b), almost all real values of nitrate concentration were within the prediction intervals of the reconstructed data. However, the nitrate prediction interval increased as the number of timestamps into the future increased. 

## 4. Discussion

Data from low-cost, in situ water quality sensors provide unprecedented opportunities to better understand spatial and temporal water quality dynamics. However, in situ sensors are prone to technical issues, which presents a challenge for the processing and analysis of environmental data. The study presented here demonstrates that it is possible to predict these missing data for reconstruction of high-frequency environmental time series using appropriate statistical methods. To our knowledge, our study is among the first to reconstruct missing nitrate data from high-frequency data collected by in situ sensors. This may be in part due to the relatively recent, standard use of such sensors for measuring nitrate concentrations in river networks and makes comparison of our findings with other studies and methods difficult. Reconstruction of high-frequency runoff data showed that a new machine learning method, “nu-support vector machines,” outperformed other machine learning methods [57]. Performance of the new method was evaluated in terms of the correlation (*R*^2^) between observed and simulated data, with their method achieving values between 0.75 and 0.95. Applying the same *R*^2^ coefficient to our simulations, we achieved *R*^2^ values between 0.976 and 0.997 when 40% of the dataset was removed and between 0.119 and 0.994 (first quartile = 0.84) when sequences of 10 days were removed, indicating that our method attains a comparatively good performance, particularly for point and short periods of missing data. Blending ARIMA forecasts and backcasts has also shown promise for reconstruction of sensor-based water quality data, including temperature, pH, specific conductance, and dissolved oxygen [58]. However, we are unable to compare our results with this work given that performance of the correction method was assessed by comparing the ARIMA-based results with corrections done manually by technicians.

Our method was able to predict all missing values present in a real-time series of nitrate concentration data. Although the prediction intervals for some predicted values were relatively wide, the median prediction interval was very low (1.34 µmol/L nitrate), indicating that many missing data had a 95% prediction interval lower than the sensor accuracy (i.e., at least 2 µmol/L) and, therefore, were precise enough for the intended use of the data. We also showed via simulation that even when 40% of the initial dataset (point observations) was missing, our method was able to accurately recover approximately 70% of the data. When day-long sequences of contiguously missing data were simulated, mimicking, for example, a persistent sensor outage or prolonged periods of quality-flagged data, performance of the method was similarly efficient. However, for week-long periods of missing data, the percentage of accurately recovered data decreased, indicating that data reconstruction is more impacted by long sequences of missing data in a row than by multiple but sporadically missing data.

Consideration of different periods of the nitrate concentration time series provided insight into the overall utility of the method and why the method may not accurately reconstruct all missing data. For example, excessive nitrate can create eutrophication issues in aquatic systems and, therefore, for the purposes of environmental management, it is important to know (i) whether the presence of high nitrate concentrations is real or anomalous, and (ii) that accurate reconstruction of the real concentrations can be achieved in a timely fashion, particularly during floods. This appears possible with the method we have developed, given that missing values during periods of sudden rises and falls in nitrate concentration were predicted accurately (Figure 3b and Figure 5c). However, when environmental covariates from co-located sensors were not available, then reconstruction relied on ARIMA, for which prediction performance was inferior to that of GAM. This finding indicates the importance of having high-frequency sensors that can collect other environmental and water quality data besides nitrate concentration at collection sites and is in accordance with results from a study of daily streamflow data [25] that found increased accuracy in the reconstruction of missing data when multiple input variables were included. 

The method presented here was developed with the objective of being able to reconstruct data that are missing, for example, due to their removal after being determined as technical anomalies from environmental data collected by high-frequency in situ sensors, using nitrate concentration data from Arikaree River. The method has currently only been applied in a binary fashion depending on the existence of covariates (other environmental data that can be used as predictors). For any one missing nitrate observation, GAM was used to predict the observation when data for all covariates were available, and ARIMA was used when data for at least one of the covariates were missing. Several avenues of study are envisaged from this work. First, future work could aim to develop a method whereby one or more environmental variables could be used as the covariate(s) according to their availability, such that ARIMA is only used when no covariate data are available. A second avenue would be to use different types of models in the framework, bearing in mind that the characteristics of time series data can influence the forecasting method that should be run. Other methods that may be suitable for the particular characteristics of water quality time series include seasonal autoregressive integrated moving average (SARIMA) for seasonal data or deep learning methods such as long short-term memory (LSTM) networks. Finally, future research could seek to confirm the applicability of our method to other sites and environmental data in order to generalize the framework.

## 5. Conclusions

Measurement errors or missing observations are recurrent and, in some cases, may reduce user perception of data quality, thereby preventing data from underpinning management actions. Here, we developed a method to successfully reconstruct missing nitrate concentration data from high-frequency in situ sensors in fresh waters, thereby adding value to the literature on anomaly detection and fulfilling a critical management need in the environmental domain. To mimic sporadically missing observations, both point data and sequences of data were removed from a two-year time series of nitrate concentration data. In 72% of cases with missing point data, predicted values were within the sensor precision interval of the original value, although the predictive ability declined when sequences of missing data occurred. The models also had stronger predictive ability when other water variables (covariates) were available. This suggests there may be advantages to deploying co-located sensors to measure covariates, even when there is a single constituent of concern, such as nitrate, by enabling a more reliable reconstruction of the nitrate time series. Our study is an important first step towards environmental data reconstruction in the information age and sets a benchmark against which future datasets and methodological developments can be compared. While we believe the general methodology presented here is generalizable to rivers in other ecosystems [59], the relationships between other water quality variables of interest may differ. Thus, future research should also focus on understanding these relationships so that co-located sensors can be optimally deployed. This will ensure that near real-time water quality data produced by low-cost in situ sensors are trustworthy and reliable enough to underpin data-enabled management decisions.

## Data Availability

Data are available through the NEON website https://www.neonscience.org/ (accessed on 4 December 2021). The National Ecological Observatory Network is a program sponsored by the National Science Foundation and operated under cooperative agreement by Battelle Memorial Institute. This material is based, in part, upon work supported by the National Science Foundation through the NEON Program.

## Appendix A

**Table A1:** NEON data. Details on sensors, variables collected, units of measurement, associated data collection intervals, and the NEON data product number for data used in this study.

Sensor	Water-Quality Variable	Unit	Published Data Resolution	Published Interval (min)	Product Number
SUNA V2	Nitrate	µmol/L	0.1	15	DP1.20033.001
YSI EXO Optical Dissolved Oxygen	Dissolved oxygen	mg/L	0.01	1	DP1.20288.001
Level TROLL 500	Water elevation	masl	0.01	5	DP1.20016.001
YSI EXO Turbidity	Turbidity	FNU	0.01	1	DP1.20288.001
YSI EXO Conductivity and Temperature	Specific conductance	µS/cm	0.01	1	DP1.20288.001
Platinum Resistance Thermometer	Water temperature	°C	0.01	1	DP1.20053.001
